# The Meaning of Volunteering among People with Severe Mental Disorders: A Phenomenological Qualitative Study

**DOI:** 10.3390/ijerph182212232

**Published:** 2021-11-21

**Authors:** Jorge Pérez-Corrales, Javier Güeita-Rodríguez, Elisabet Huertas-Hoyas, Cristina García-Bravo, Romain Marconnot, Carmen Jiménez-Antona, Juan Francisco Velarde-García, Domingo Palacios-Ceña

**Affiliations:** 1 Research Group of Humanities and Qualitative Research in Health Science (Hum&QRinHS), Department of Physical Therapy, Occupational Therapy, Physical Medicine and Rehabilitation, Universidad Rey Juan Carlos Avenida Atenas s/n, 28922 Alcorcón, Spain; jorge.perez@urjc.es (J.P.-C.); romain.marconnot@urjc.es (R.M.); carmen.jimenez@urjc.es (C.J.-A.); domingo.palacios@urjc.es (D.P.-C.); 2 Research Group in Evaluation and Assessment of Capacity, Functionality and Disability (TO+IDI), Department of Physical Therapy, Occupational Therapy, Physical Medicine and Rehabilitation, Universidad Rey Juan Carlos, Avenida Atenas s/n, 28922 Alcorcón, Spain; elisabet.huertas@urjc.es (E.H.-H.); cristina.bravo@urjc.es (C.G.-B.); 3 Department of Nursing, Instituto de Investigación del Hospital Gregorio Marañón (IiSGM), Red Cross College, Universidad Autónoma de Madrid, Calle Reina Victoria 28, 28003 Madrid, Spain; jvg@cruzroja.es

**Keywords:** vocational rehabilitation, volunteers, work, mental disorders, qualitative research, stigma

## Abstract

This study aimed to describe the perspectives of people with severe mental disorders who volunteer regarding the relationship between volunteering and work, from the framework of personal recovery. A qualitative phenomenological study was undertaken. Purposive sampling was conducted on people with severe mental disorders who participated in volunteering. In-depth unstructured and semi-structured interviews were used, during which researchers took handwritten field notes. An inductive thematic analysis was applied. Twenty-three participants with severe mental disorders were included (16 men and 7 women) with a mean age of 47 years. Three themes emerged: (a) the relationship between volunteering and working; (b) thinking about a possible future job; and (c) disclosing a mental health condition. Volunteering is perceived as a substitute to working, although not all participants feel able to work, and they do not always disclose that they suffer from a mental health condition.

## 1. Introduction

The concept of recovery in the area of people with severe mental disorders (SMD) (a diagnosis of non-organic psychotic disorder such as schizophrenia, schizoaffective disorders or bipolar affective disorder, among others, with a severe dysfunction of global functioning) clashes with the established belief that it is impossible to have a positive evolution in the course of certain mental disorders, such as schizophrenia, which is considered chronic and disabling [1].

As the existing literature has shown [1,2,3,4,5], the definition of recovery is complex, and the lack of consensus leads to the existence of multiple conceptualizations. The internationally accepted approach [1,2,3,4] is the terminological clarification made by Davidson and Roe [5] which differentiates between the clinical recovery or “recovery from the disease” arising from the knowledge and clinical-professional research, and the subjective recovery, personal recovery or “being in recovery”, derived from the social movement of the people who have a diagnosis of mental disorder and who claim their right to self-determination and their inclusion in the community. This paradigm is considered to be the main theoretical and practical framework in the field of mental health in the last 30 years [2].

The involvement of people with SMD in productive activities such as volunteering or employment allows them to assume responsibilities and control over their own lives. These are essential elements of the recovery process, leading to empowerment and inclusion in the community [3,6,7]. Therefore, within the framework of personal recovery, both employment and volunteering are not only sought-after goals, but also ways of recovering the connection with the community, the hope of continued recovery, the acquisition of a personal and social identity, and the feeling of empowerment [3,6].

People with SMD claim to have a job or volunteer, yet this is an unmet need [8]. People with SMD appreciate engaging in productive activity that facilitates their participation in society. However, these individuals also have high rates of unemployment and difficulties both accessing and holding a job [7,9]. Consequently, the paid employment rates of people with SMD, and especially of people who are within the schizophrenia spectrum, is around 10–20%, meaning that this population is in a situation of vulnerability and risk of social exclusion [7].

In Spain, despite the legislation [9,10] that regulates and protects the inclusion of people with disabilities associated with mental health problems in ordinary and protected employment, the population of people with mental disorders have the lowest employment rate, with just 16.9%, whereas the employment rates of people with disabilities is 25.9%, and that of people without disabilities is 66.9% [11].

Recently, Charette-Dussault and Corbière identified barriers to employment for people with SMD, such as lack of experience, cognitive impairment, long periods of unemployment, social stigma, low perceived self-efficacy, and low perceived control over one’s own life [12].

In Spain, people who have been granted total or absolute permanent invalidity can work in paid employment with the authorization of compatibility determined by the National Institute of Social Security (NISS) [13]. Depending on each particular case, they may combine the receipt of the subsidy with the salary from the job, or, alternatively, stop receiving the subsidy [13]. This situation may be beneficial for people with SMD as a way to return to work; however, the NISS may also consider that the reason for the granted incapacity for work situation has been resolved and the person loses the benefit [13].

The vocational goals of people with SMD are variable, as many prefer to engage in training or volunteering rather than work [14]. There is a growing interest in vocational rehabilitation programs [15,16], and volunteer work is considered a fundamental element in the recovery process [17,18] and a stepping-stone to employment. This would allow these people to acquire the necessary skills to successfully pursue a competitive job [15,19]. Held and Lee described volunteering as a promising strategy within interventions with people with SMD, indicating that, despite the benefits shown by volunteering in the general population, limited research is available regarding the positive results of volunteering among people with SMD [20].

The aim of our study was to explore the perspectives of people with SMD on volunteering and employment.

## 2. Materials and Methods

### 2.1. Design

A qualitative [21] phenomenological [22] study was conducted to describe the experiences and perspectives of people with SMD on volunteering, and to analyze the relationship between volunteering and working for people with SMD who volunteer. The use of phenomenology is necessary to study the experiences and individual perspectives of people in the face of certain situations, events and phenomena, such as illness or disability [21,22].

### 2.2. Research Team

The research team was comprised of three woman and five men, including three occupational therapists (JP-C, EH-H and CG-B), two research nurses (DP-C and JFV-G), a physical educator (R-M), and two physical therapists (JG-R, CJ-A). Three members had clinical and research experience with people with SMD; and seven members had experience in the application of qualitative studies in health sciences and mental health. None of the investigators had previous contact with any of the study participants. Prior to the start of the study, the positioning of the investigators was established based on their beliefs, previous experience, theoretical framework, and motivation for the research [21,23]. The researchers used the recovery paradigm, [19] understanding that for people with SMD, recovery is a dynamic process, supporting the ability to regain control over their lives and their own decisions (empowerment), assigning meaning to their lives, fostering the development of a new identity as opposed to the “mentally ill” identity, helping them face the future with hope, and strengthening their connection with others [3].

### 2.3. Participants and Setting

The participant recruitment procedure involved contacting different entities of the community mental health services of the Autonomous Communities of Madrid and Catalonia (Spain). Purposive sampling was used [21]. Recruitment took place until redundant information was obtained from the data [21].

The inclusion criteria consisted of: (a) people aged between 18 and 65 years, (b) with a diagnosis of non-organic psychotic disorder (F20.x, F21, F22, F24, F25, F28, F29, F31.x, F32. 3 and F33) according to the International Classification of Diseases, 10th revision (ICD-10) [24]; (c) a course of ≥2 years with the mental illness; (d) moderate to severe global functioning dysfunction with scores of ≤ 70 on the global assessment of functioning scale [25]; (e) participation in a volunteer program, based on the regulations on volunteer activities in Spain [26]; (f) the participant had to maintain all legal rights and autonomy; and (g) all participants had to sign the informed consent. The exclusion criteria included: (a) participants with acute psychopathological imbalance, and (b) people with disorders affecting language or comprehension. The research team established the inclusion and exclusion criteria for study participants based on their clinical experience and review of scientific studies. Subsequently, they transferred these criteria to the mental health service professionals, who selected those participants who met the inclusion criteria and put them in contact with the researchers.

### 2.4. Data Collection

Data were collected (JP-C) between September 2016 and April 2017. The first stage of data collection consisted of unstructured interviews (participants 1–11), based on the following questions: What type of volunteering do you do? What is your experience of volunteering? After the initial data collection, new areas of interest were identified from the participants’ perspectives. During the second stage, a semi-structured interview guide (Table 1) was developed, incorporating data from the first phase (participants 12 to 23) in order to provide a deeper understanding of the participants’ experiences [16]. In addition, during the face-to-face interviews, researchers used prompts: (a) to encourage participants to provide more details (‘Can you tell me a little more about that?’), (b) to encourage participants to continue talking (‘Have you experienced the same thing since then?’), and (c) to resolve confusion (paraphrasing something the participant had said). Twenty-three interviews were conducted (one per participant). The mean duration of the interviews was 66 min (S.D. 9.28) during the first phase, and 61 min (S.D. 16.67) in the second phase. In total, 1459 min of interviews were audio-recorded (729 min in the first phase and 730 min in the second phase). Twenty-three field notes were collected from the researchers. All interviews were conducted by JP-C on the premises of community mental health services. Nobody else was present during data collection.

### 2.5. Data Analysis

The in-depth interviews were transcribed together with the researchers’ handwritten field notes made during the interviews [21]. Interviewers take handwritten notes to document a wide range of information, including: casual and structured observations; verbatim quotes; paraphrases of participant responses; interview backup documentation; the researcher’s questions; questions, conclusions, and observations discussed during the data collection process. An inductive thematic analysis was performed [27]. During coding, the most descriptive content was identified (significant units); subsequently, the units with similar content were grouped into thematic code groups. Finally, the themes describing the participants’ experiences of volunteering were identified. Three researchers (JP-C, DP-C, JG-R) conducted the analysis independently, and compared and cross-checked the results in analysis meetings, where the final results were identified. In cases of differences of opinion, identification of themes and data saturation or information redundancy was decided by consensus. No qualitative software was used.

### 2.6. Quality Criteria

The study was performed according to the Consolidated Criteria for Reporting Qualitative Research (COREQ) [28] and the Standards for Reporting Qualitative Research (SRQR) guidelines [29]. In addition, the Lincoln and Guba criteria were used, which include the control of credibility, transferability, reliability and confirmability of the data [30]. The following techniques were used: (a) triangulation of data collection instruments and triangulation of researchers during analysis (credibility); (b) validation of participants (credibility); (c) comprehensive description of the study (transferability); (d) provision of details of researchers’ and participants’ characteristics (transferability); (e) records of the reflexivity process during the study (confirmability); (f) coding based on participants’ narratives (credibility and confirmability); and (g) external audit (reliability) [30].

### 2.7. Ethical Considerations

This study was approved by the Clinical Research Ethics Committee at the Rey Juan Carlos University (code: 040220160616). In addition, permission was obtained from Fundación El Buen Samaritano, Grupo Exter S.A., Instituto de Neuropsiquiatría y Adicciones del Parc de Salut Mar, and Hermanas Hospitalarias. In all cases, informed consent and permission to record the interviews was obtained from the participants.

### 2.8. Patient and Public Involvement

Firstly, the researchers informed all the potential participants of the study design to enable an informed choice about their decision to participate in the study. The study design was pre-established, thus the participants were required to adhere to this design. Second, participants involved in the study helped researchers identify the research questions in order to create a semi-structured question guide. The questions of the semi-structured interviews were based on unstructured previous interviews where the participants were able to describe their experiences and perspectives without limitations and to develop their own relevant content. In addition, participants were involved in confirming the data obtained at the various stages of data collection and analysis. All participants were offered the opportunity to review the audio or written records, as well as the subsequent analysis to enable the interpretation of their experiences by the researchers. All participants were given an audio copy of the interview; however, none of the participants made additional comments. To verify the analysis performed for each interview, half of the participants were contacted, all of which confirmed the analysis performed by the researchers. The results of this study will be disseminated to participants via email by providing participants with the open access link for the study. In addition, the authors will disseminate the results of the study via presentations at national and international conferences, and these findings will be communicated to community mental health services.

## 3. Results

Twenty-three participants with SMD were included (16 men and 7 women) with a mean age of 47 years (SD 9.21) and a mean evolution of the disorder since diagnosis of 17.91 years (SD 8.23). All participants were receiving permanent disability benefits. They performed different types of volunteering in coordination with different social entities through the community mental health services: supporting other people with SMD, volunteering with animals, caring for older people, library assistants, chess teachers with children at risk of social exclusion, soccer coaches with children with intellectual disabilities, and food distribution to families at risk of social exclusion. There were no dropouts in the study.

Three specific themes emerged: (a) the relationship between volunteering and working; (b) thinking about a possible future job; and (c) disclosing a mental health condition.

### 3.1. Theme 1. The Relationship between Volunteering and Working

Participants described how volunteering made them feel like they were employed in a job, as they were expected to fulfill schedules, rules, responsibilities and obligations. Volunteering kept them active and led to feelings of empowerment, even when volunteering involved short periods (hours or days a week). 

Most participants viewed volunteering as a job or a substitute for work, although less demanding or intense and unpaid. In relation to a conventional job, the participants felt that the demands and responsibilities constituted the main barriers to feeling capable for working or being able to maintain a job:

“An obligation, a commitment, knowing that you have to get up on time, that it is like a job. Some people go to work in a factory or an office or whatever they want, and you go to take some granddads out, it’s as if you were a caregiver, but without being paid.” (participant 9); “I wanted to work, but maybe I would screw something up working, because then it becomes an obligation, even if I earn some money, it’s an obligation and it’s not the same as volunteering. At work, even if you have a disability, I suppose they are going to demand a little bit more from you.” (participant 15).

Two deviating cases were noted (participant 16 and participant 18), because in the past they held jobs protected by public agencies. These are jobs that are paid and are reserved for the integration of people with disabilities. Participant 16 reported how sometimes the person with SMD is paid less for doing the same work as a “healthy” person. At other times, it requires the person to force themselves to work harder to avoid others perceiving their mental condition: “just because you are sick, you are not going to be inferior to the person who is healthy. Because maybe they put you in a protected job and you work twice as much as the other person. And they are paying you less. If you are sick, you have to work harder so that the boss doesn’t notice that you’re sick.” (participant 16). Furthermore, participant 18 explained that in order to receive the same salary that he obtained from his pension (without having to work), he had to work long hours and it was not worth it. He concurred with participant 16 that there was a certain degree of discrimination against people with SMD who work, since they have to work long hours with a reduced income. Participant 18 felt more comfortable volunteering than working in a protected and more recognized job: “I worked a lot for the same salary I get from the absolute disability and I didn’t feel comfortable because they put a lot of pressure on me. There is a lot of pressure on us in protected jobs. I prefer not to get paid at all and be a volunteer. Those of us who are mentally ill are made to work long hours and then they take money out of our paychecks. I feel more included in these volunteer jobs than when I was working earning money.” (participant 18).

### 3.2. Theme 2. Thinking about a Possible Future Job

Some participants acknowledged that participating in volunteering gave them hope that in the future they would be able to return to work or feel more confident. Volunteering is a way to test themselves and a way to prepare for the world of work. Thus, volunteering is a “pre-work” rehearsal that allows them to gain experience. Performing well during volunteering makes them feel more confident, optimistic, and envision the transition to a job: “I am more confident than before I began volunteering, now with the volunteering I feel confident that I can work.” (participant 6); “If I manage to do almost everything this year as a volunteer and that goes well, then maybe next year I can look at working for four hours. The last 10 or 15 years that I have left, for that, even if I have the pension, but I can continue working which makes me feel better.” (participant 15).

Receiving compensation or a pension is a relevant element to consider in their future employment, and the participants were hesitant between choosing to work even if they received a pension, or not working because they had a pension that covered their needs and they had economic stability: “At the beginning I said ‘I don’t want to work anymore in my lifetime’, and now, through volunteering, my mindset has changed (…) but watching my back, if, say, tomorrow I get a job, I’m not going to let them take away my allowance. Because to earn less money than what I’m earning with my allowance, I’d rather not work.” (participant 19).

### 3.3. Theme 3. Disclosing a Mental Health Condition

Finally, there were different perspectives on how to communicate their mental condition. Not all volunteering entities were aware of the participants’ condition. Participants were hesitant whether or not they should publicly share their situation as this might negatively influence their relationship with other people when volunteering. Although all participants preferred to keep a low profile, they said they would not mind disclosing their situation if this arose in the context of an informal conversation: “We don’t tell anyone that we are mentally ill, when you volunteer there are a lot of people, and we don’t have to. But if they ask and the conversation arises ‘hey, well, yes… look, I have a mental illness’. But there is no reason to label yourself.”(participant 2); “They don’t know about the disease we have... I don’t think it had an influence. My colleague told me that they don’t talk much about the disease we have. If they knew? Well, maybe they wouldn’t want to [continue receiving volunteer help] or maybe they would. I don’t know.” (participant 8).

## 4. Discussion

The results of our study are in line with previous studies [31,32], where people with mental disorders identified volunteering as a means that provided them with a sense of organization, offered them the opportunity to acquire responsibility and balance in their lives, and provided meaning related to job satisfaction. 

Some of our participants described how participating in volunteering has encouraged them to consider future employment. Fegan described how people with SMD also identified volunteering as a rehearsal or preparation for a future job, since they were contributing positively as volunteers and could later do so as workers [32].

There is a growing interest in vocational rehabilitation programs and interventions in the workplace as part of the recovery process for people with SMD [15,16]. From this perspective, volunteering is a preliminary step to employment, which enables the acquisition of skills for subsequent incorporation into a job [15,19]. In contrast, other models, such as the Individual Placement and Support model, directly incorporates the person with SMD into a job, receiving the necessary support within the job itself. This model has been shown to be more effective in achieving employment than the use of volunteerism as pre-vocational skills training or classical vocational rehabilitation [33,34]. These results have also been described in long-term unemployed people among the general population, where it has been identified that volunteering is not an adequate means for obtaining employment, although it does lead to greater self-respect and self-esteem [35].

Our findings highlight that some participants felt that they would not be able to work because of the demands of competitive employment and therefore they preferred to volunteer. Participants in the Prior et al. [36] study had a low perception of their own work skills, in particular their ability to cope with the pressure of work and with keeping a daily work schedule and working day. In addition, Hielscher and Waghorn showed that despite the strong value that people with mental disorders place on employment, self-stigma is one of the main barriers limiting their search for employment [37]. The “Why Try” effect has been described as a direct consequence of self-stigma whereby people stop trying to achieve their life goals such as seeking a job due to the perceived low self-efficacy that conditions their belief of feeling incapable of performing and maintaining a job [38,39,40,41].

In our study, different participants described the allowance received from their pension as an element to be considered in their decision to return to work. The pension as a factor related to job seeking by people with SMD was described by Saavedra et al. [42]. These authors described that, although it was not a deterrent, there were participants who expressed the fear of being left with nothing if they started working and subsequently lost their job. However, in Spain, people who have been granted total or absolute permanent incapacity can supplement their pension payments by receiving a salary from employment, although in some cases this subsidy may be lost [13]. Similarly, Fegan showed how people with SMD are afraid of losing the economic benefits obtained through their pension [32]. In Spain, the economic crisis and job insecurity have been described as elements that might make employment not synonymous with independence, both in people with SMD and in the general population [42]. Along the same lines, social factors have also been identified in the review on barriers to job acquisition in people with SMD by Charette-Dussault and Corbière [12]. According to these authors: “It is understandable that people with severe mental illnesses are reluctant to leave these programs for a job, often at a minimum wage and of uncertain security” (p. 535), referring to different interventions where these people acquire pre-employment skills, and recommending the need for political and social changes to resolve this situation. In contrast, for the participants in the study by Hanisch et al. on perceived barriers to working in people with mental disorders, the loss of disability-related economic benefits was not a major problem [14].

Finally, our results reveal different perspectives on whether or not people should disclose their mental condition when volunteering, because this information could impact their relationship with peers. The possibility of people with SMD being stigmatized by society has been identified in multiple studies [43,44,45] as one of the most important concerns. It is also perceived as a major barrier to recovery and is identified as being especially painful when shared with people who are close to the patient [46]. The ambivalence stated by the participants in our study in relation to disclosing their status was also identified by Soeker et al. in the work setting [47]. These authors described how disclosing their condition increased their anxiety due to the fear of being stigmatized due to the poor social reputation of this group (incapacity or inefficiency), although, conversely, they could also receive more support, which would improve their job performance [47]. At the community level, Gunnarsson and Eklund described how people with SMD could be stigmatized if during their participation in community activities, someone associated them with users of the mental health center [48]. McGuire et al. identified that stigma and critical comments made people with SMD feel judged and treated as mentally ill and not as “normal” people [43].

The first limitation is that the results cannot be extrapolated to all people with SMD who participate in a volunteer program due to the limitations of the qualitative methodology [21]. However, in qualitative research it is possible for qualitative results be transferred to other contexts or settings with other participants. This trustworthiness criteria is called transferability which is facilitated by the researcher who provides thick description, i.e., sufficient detail to allow readers to make an informed judgement about whether they can transfer the findings to their own situation [30,49]. The second limitation is related to the recruitment of the participants, as people with SMD who were immersed in a process of work rehabilitation were not included, and therefore their perceptions may be different. Future lines of research may involve qualitative studies that delve deeper into the lived experiences of people with SMD who support other people with SMD in the recovery process, both as volunteers and in the workplace.

Finally, we believe that these findings can help professionals to understand how people with SMD experience volunteering, their relationship with the work environment and the impact that volunteering has on the recovery process, from their perspectives. This knowledge can contribute to promote these interventions in the different health and/or social care services, both hospital and community-based, in order to support the recovery of people with SMD and their inclusion in the community.

## 5. Conclusions

For people with SMD, volunteering has certain similarities to the work environment, such as compliance with schedules, rules and responsibilities. Not all participants feel qualified to work; however, for some individuals, volunteering represents an initial ‘rehearsal’ that has awakened their interest in pursuing a paid job. There are doubts about whether or not to disclose their mental condition while volunteering, since this may have repercussions, and therefore, people with SMD prefer to remain unnoticed.

The implementation of volunteer programs from community mental health services can contribute to the empowerment and inclusion in society of people with SMD. Mental health professionals must contribute to the creation of spaces where people with SMD can feel useful and assume responsibilities, as a previous step to labor inclusion or as an end in itself. Volunteering can be included in vocational rehabilitation programs and work rehabilitation services as an element of motivation prior to employment or as an experience that contributes to the recovery process.

## Figures and Tables

**Table 1:** Semi-structured question guide.

Opening Questions	What Type of Volunteering Do You Do? What Is Your Experience as a Volunteer?
Volunteering and work	What are the similarities and differences between volunteering and paid employment? What factors influence you to volunteer rather than pursue paid employment? Do you see volunteering as a responsibility, an obligation and/or a commitment? What is this like for you? How does volunteering influence your future job prospects? Where you volunteer, do they know that you have a mental disorder? What are the motivations for deciding whether or not to disclose this information?

## Data Availability

The personal data related to this study is stored in the data protection file belonging to the Rey Juan Carlos University. Considering the qualitative nature of this study, we are unable to provide the transcribed files, in compliance with the Spanish Protection Data and Information Act (1999). Data analysis was performed manually. No qualitative analysis software was used.
